# Left Ventricular Mass Index Predicts Renal Function Decline in Patients with Chronic Kidney Disease

**DOI:** 10.3390/medicina60010127

**Published:** 2024-01-10

**Authors:** Antonio Lacquaniti, Fabrizio Ceresa, Susanna Campo, Francesco Patané, Paolo Monardo

**Affiliations:** 1 Nephrology and Dialysis Unit, Papardo Hospital, 98158 Messina, Italy; ant.lacq@gmail.com (A.L.);; 2 Department of Thoracic and Cardiovascular Surgery, Papardo Hospital, 98158 Messina, Italyf_patane@hotmail.com (F.P.)

**Keywords:** left ventricular mass index, brain natriuretic peptide, chronic kidney disease progression, natriuretic peptides

## Abstract

*Background and Objectives*: Several studies revealed a relation between abnormal cardiac remodeling and glomerular filtration rate (GFR) decline, but there are limited data regarding echocardiographic changes in chronic kidney disease (CKD). This study evaluated the abnormal cardiac structures characterizing patients with CKD, assessing the independent association between echocardiographic parameters and the risk of decline in renal function. *Materials and Methods:* In total, 160 patients with CKD were studied. All patients underwent an echocardiographic exam and 99mTc-DTPA renal scintigraphy to measure the GFR. After the baseline assessments, patients were followed prospectively for 12 months, or until the endpoint achievement, defined as a worsening in renal function (doubling of baseline serum creatinine, GFR decline ≥25%, the start of dialysis). *Results:* Patients with GFR values of 34.8 ± 15 mL/min, identifying stages III–IV of CKD, were associated with high levels of left ventricular mass index (LVMi) (101.9 ± 12.2 g/m^2^), which was related to proteinuria, systolic blood pressure, and pulmonary artery systolic pressure in a multiple regression model. During the observational period, 26% of patients reached the endpoint. Regression analysis revealed LVMi as a predictor of change in renal function after adjusting for kidney and cardiac risk factors. Multiple Cox regression indicated that an increase in LVMi was associated with a 12% increased risk of kidney disease progression (HR: 1.12; 95% CI: 1.04–1.16; *p* = 0.001). *Conclusions:* In patients with CKD, high LVMi represents an independent predictor of the progressive decline of the renal function, until the start of renal replacement therapy. Echocardiography can help identify patients at high risk for renal disease worsening in patients with CKD independently of clinical cardiac involvement.

## 1. Introduction

Patients with chronic kidney disease (CKD) present an elevated cardiovascular risk, frequent hospitalizations, and higher rates of morbidity and mortality related to cardiac disease [1]. The interaction between the cardiovascular and renal systems depicts cardio-renal syndrome, with different types according to the site of the initial injury and the acute or chronic nature of the process [2]. This crosstalk is transferred into anatomical changes in the heart, determining abnormalities in structure and function, observed even if minimal renal damage occurs [3,4]. These conditions should be referred to subclinical settings, not determining symptoms, and preceding the clinical presentation of heart failure, including greater left ventricular mass (LVM), greater pulmonary artery systolic pressure (PAPs), and lower left ventricular ejection fraction (LVEF).

Data from the Chronic Renal Insufficiency Cohort (CRIC) Study revealed a close interaction between cardiac structure and the risk of renal failure in a population with CKD, hypothesizing that cardiac abnormalities and renal damage are based on the same causal pathways, related to the interaction between hypertension, heart failure, and coronary artery diseases [5]. Furthermore, other prognostic evidence has been assessed among adults with CKD, revealing a causal link between adverse cardiac remodeling and increased risk of heart failure-related morbidity and mortality [6].

These geometric alterations, such as left ventricular hypertrophy and an increased left ventricular mass index (LVMi), primarily arise as a maladaptive response, compensating for a volume and pressure overload. In this context, and closely linked to these structural abnormalities, natriuretic peptides (NPs) try to regulate salt and water balance and volume homeostasis. Once released, these peptides act on kidneys, blood vessels, and adrenal glands, leading to natriuresis, diuresis, and vasodilation. The final effect of both atrial (ANPs) and brain (BNPs) natriuretic peptides is to compensate for these alterations, promoting cardiovascular and renal protective actions, and inhibiting mesangial cell proliferation and renal fibrosis [7,8,9]. This adaptive mechanism for the volume and pressure overload results in left ventricle hypertrophy and dilatation, increasing the interstitial space, and leading to myocardial fibrosis, explaining the subclinical systolic dysfunction.

Echocardiography can identify these abnormalities typically associated with kidney dysfunction, highlighting a precocious abnormal left ventricular geometry or high LVMi, regardless of the ejection fraction [10].

Whereas several studies revealed a relation between abnormal cardiac remodeling and glomerular filtration rate (GFR) decline [11,12], there are limited data regarding echocardiographic changes in populations with CKD. The timing over which cardiac remodeling occurs in CKD, the reversibility, or whether echocardiographic changes are associated with cardiac-related morbidity and mortality remains unknown. The AASK study addressed this issue in African American patients with pre-existing non-diabetic hypertensive CKD, showing that LVMi was not associated with a combined outcome of doubling of creatinine or end-stage renal disease [13]. Conversely, a retrospective analysis, also evaluating an African American population, showed a significant association with LVMi, but not LVEF or PAPs, with a composite outcome of estimated GFR decline > 30% or progression to end-stage renal disease [14].

This study aimed to evaluate the abnormal cardiac structures characterizing patients with CKD and assess the independent association between changes in echocardiographic parameters and subsequent risk of decline in renal function in a population with CKD.

## 2. Patients and Methods

### 2.1. Patient and Study Design

In total, 160 patients with CKD, followed at the Nephrology Unit, Papardo Hospital, Messina, Italy, were involved; the enrollment phase started in January 2021. All patients had 3–4 CKD stages, according to the guidelines of the Kidney Disease: Improving Global Outcomes (KDIGO) [15].

Renal function measurements were performed at baseline using renal scintigraphy, with the administration of the radiopharmaceutical 99mTc-DTPA. An inclusion criterion was a clinically euvolemic status, without symptoms of acute or chronic cardiac disease. All patients had normal BNP levels at the enrollment, limiting confounding by cardiac dysfunction or volume overload. Patients with serum creatinine above 6 mg/dL and/or GFR < 15 mL/min, severe proteinuria (>3 g/d), acute inflammatory states, and treatment with steroids or immunosuppressors were excluded from this study. Patients with severe heart failure defined by a left ventricular ejection fraction <50% or regional wall motion deficit assessed by echocardiography were not included. The local Ethics Committee of the University of Messina approved this study, conducted according to the guidelines of the Declaration of Helsinki, and all patients provided written informed consent.

### 2.2. Echocardiography

All patients underwent an echocardiographic exam, performed using the Vivid 7, GE (Vingmed Ultrasound AS, Horten, Norway).

End-diastolic(d) and end-systolic(s) left ventricular (LV) internal diameters (LVIDs), inter-ventricular septum thickness (IVS), and posterior wall thickness (PWT) were measured from two-dimensionally guided M-mode. LVM index (LVMi) was estimated by using the corrected American Society of Echocardiography method [16]. Left ventricular (LV) end-diastolic volume (LVEDV) and end-systolic volume (LVESV) were calculated using the Teichholz correction of the cube formula [17].

The pulmonary artery systolic pressure (PAPs) or systolic right ventricular pressure was calculated using the Bernoulli equation [18]. Pulmonary hypertension (PH) was defined as PAPs ≥ 35 mmHg [19]. Mitral inflow velocity was also traced and the peak early (E) and late (A) trans-mitral flow velocities, and the ratio of early to late peak velocities (E/A) were derived. E/A ratio less than 0.75 and more than 1.8 was considered diastolic dysfunction.

### 2.3. Prospective Follow-Up

The study endpoint was defined as worsening in renal function identified by a doubling of baseline serum creatinine, an accepted surrogate index of GFR slope [20] or ≥25% decrease of baseline measured (m) GFR or end-stage renal disease (ESRD) requiring dialysis. After the GFR decline assessment and/or a first doubling of creatinine, the latter was repeated after three weeks to confirm the value, ruling out potential acute kidney injury. During a 12-month follow-up period, GFR was evaluated through the CKD-EPI formula [21].

### 2.4. Statistical Analyses

Differences between groups were established by unpaired *t*-test or by ANOVA followed by Bonferroni’s test for normally distributed values and by Kruskal–Wallis analysis followed by Dunn’s test for nonparametric values. Correlation coefficients were used as appropriate to test correlations between LVMi and other variables. For the analysis of LVMi as a predictor of worsening in renal function, we performed a univariate regression analysis with baseline LVMi as the independent variable and worsening in renal function (doubling of serum creatinine and/or ESRD) as a dichotomic dependent variable. Subsequently, this association was adjusted for covariates that potentially could be confounders using multivariable regression models, built stepwise. The association was adjusted for sex and age (model 1), for covariates that are causally linked to CKD progression [22] (model 2), and for cardiologic risk factors (model 3). Adjusted risk estimates for CKD worsening were calculated using univariate followed by multivariate Cox proportional hazard regression analysis. Based on reported correlations between BNP and GFR [23], a sample size of 140 to 150 patients was considered sufficient to achieve statistical significance with 85% power and an allowable two-sided error of 0.05.

## 3. Results

### 3.1. Patients Baseline Characteristics

The baseline clinical characteristics of 160 patients with CKD are shown in Table 1.

Mean serum creatinine was 2.01 ± 1 mg/dL, with a mean mGFR of 34.8 ± 15 mL/min (IQ range 20–44 mL/min). According to baseline stages of GFR, 41 patients belonged to stage II, whereas 76 and 43 patients were included in stage III and IV, respectively. Baseline BNP ranged from >10 to 86 pg/mL (median (IQR) 18.7 (10–32.2) pg/mL).

### 3.2. Echocardiographic Data

IVST and PWT increased in parallel with the severity of kidney dysfunction. The inclining stage of renal failure was associated with a significant increase in LVMi (101.9 ± 12.2 g/m^2^), as well as a systolic function defined by the LVEF parameter, which remained within normal limits in all patients, but a noticeable tendency to decrease with a decline in mGFR was observed. Furthermore, PH was detected in 25% (41/160) of patients overall. In particular, 9/41 patients (22%) had a GFR ≥ 60 mL/min, whereas the remaining subjects belonged to stage III (17/76, 39%) and IV (15/43, 35%). The mean value of PAPs was ≥35.8 ± 9.2 mmHg, ranging from 25 to 50 mmHg.

### 3.3. Univariate Correlations and Multiple Regression Analysis

At baseline, LVMi was directly correlated to systolic blood pressure (sBP), BNP, PAPs, proteinuria, and natriuria, whereas it was inversely correlated with hemoglobin and mGFR values. Using LVMi as a dependent variable in a multiple regression model, the associations with sBP (β = 0.61, *p* = 0.0001), natriuria (β = 0.76, *p* < 0.001), proteinuria (β = 0.70, *p* = 0.001), and PAPs (β = 0.56, *p* = 0.01) remained significant, while those with BNP and hemoglobin disappeared (Table 2).

### 3.4. Progression Endpoint during Follow Up

During the observational period, 41 patients (26%) reached the endpoint. In particular, 12 patients required renal replacement therapy, whereas 29 patients experienced a doubling of baseline serum creatinine, without needing dialysis treatment. All 12 dialyzed patients belonged to stage IV, at the enrolment phase, whereas the remaining 29 patients belonged to stages IIIb and IV. The 119 patients (74%) who did not experience kidney disease progression completed the whole observational period. Table 1 displays the main data and statistical differences between patients with or without kidney disease progression during the follow-up period. The “progressors” and “non progressors” groups were well matched for the major CV risk factor.

Furthermore, seven patients who experienced a major cardiovascular event during the year of follow up, had the highest LVMi observed (122.1 ± 9.7 g/m^2^), such as the PAPs values (54.8 ± 5.1 mmHg). In particular, all these patients belonged to the progressor group, and 2 of them underwent hemodialysis.

For the analyses of LVMi as a predictor of decline in renal function during the 1-year follow-up period, we performed regression analysis with baseline LVMi as the independent variable and doubling of serum creatinine, 25% reduction in baseline mGFR, and/or ESRD requiring dialytic treatment as a dependent variable. This association was significant after adjusting for sex and age (model 1; *p* < 0.001), after an additional adjustment for kidney (model 2; *p* = 0.003) and cardiac risk factors (model 3; R2: 16.2; adjusted OR: 1.07; 95% CI: 1.02–1.12; *p* = 0.01), as reported in Table 3.

### 3.5. Univariate/Multiple Cox Regression Analysis and Incidence of the Decline in Renal Function

To identify putative risk factors associated with the incidence of the decline in renal function, we performed a Cox regression analysis. Univariate analysis showed that uric acid, LVEF, LVMi, PAPs, mGFR, proteinuria, and BNP values were significantly associated with the endpoint. Multiple Cox regression indicated that an increase in LVMi was associated with a 12% increased risk of kidney disease progression (HR: 1.12; 95% CI: 1.04–1.16; *p* = 0.001), representing one of the main risk factors for the progression of kidney disease, among the variables analyzed. Table 4 summarizes the data from these analyses.

## 4. Discussion

This study demonstrates that high levels of LVMi characterize patients with CKD, predicting the worsening in renal function and providing prognostic information in addition to well-established renal risk markers, such as proteinuria. In fully adjusted models that accounted for baseline clinical characteristics and echocardiographic parameters, increased LVMi was independently associated with a high risk of renal function decline. Left ventricular mass occurs early in the course of renal disease, in association with hypertension and mild fluid overload, and then further significant changes occur with more marked renal impairment, in the pre-dialysis phase [24].

The data obtained through cardiac magnetic resonance revealed that LVMi was the earliest changed parameter during the development and progression of CKD, which occurred when the GFR began to drop. In particular, in patients with GFR < 30 mL/min/1.73 m^2^, LVMi was independently correlated with ventricular volume indexes, indicating that LV hypertrophy is the bridge between CKD and increased cardiovascular disease [25].

Our data confirm these connections, revealing a relation between LVMi, proteinuria, and systolic blood pressure, but an increased LVMi was an independent predictor and risk factor of decline in renal function.

This marker reflects an increased afterload, secondary to hypertension or calcific arteriosclerosis. Concomitantly, it indicates an increased cardiac preload due to salt and water retention, conditions that typically characterized a CKD patient. Moreover, the patient with nephropathy is more complex, due to subclinical and chronic inflammation associated with a marked endothelial dysfunction, pro-coagulant state, and oxidative stress; the final events are ventricular hypertrophy and diffuse cardiac fibrosis, which are highlighted by an increased LVMi.

Similar data have been assessed in more than two thousand non-dialyzed patients with CKD in whom LVMI and ratio of high-density lipoprotein and C-reactive protein were independently correlated with renal disease progression [26].

Similarly, LVMI assessed via echocardiography represented a potential prognostic factor in patients with IgA nephropathy, underling a correlation between high LVMI values, renal replacement therapy, and cardiovascular events [27].

However, our data refer to patients with an euvolemic status, without symptoms of acute or chronic cardiac disease and with normal BNP levels at the enrolment, limiting confounding bias by pre-existing cardiac dysfunction or volume overload. This strict selection suggests that renal disease induces subacute and asymptomatic cardiac damage over time, activating a vicious cycle with initial compensative mechanisms that will be the physiopathological cornerstone of the cardio-renal syndrome. Moreover, these findings promote the use of echocardiograms in patients with nephropathy to detect cardiac remodeling, even if patients do not refer to specific cardiac symptoms at the early stages of CKD.

This study also focused on two other parameters that should be included in the routine management of patients with nephropathy: BNP and PAPs levels.

Firstly, BNP did not influence these results, confirming that kidney dysfunction, alone, is not sufficient to increase its levels. Several reports in which low and/or normal BNP levels were observed in patients with reduced GFR confirmed our data [28,29].

Rather, BNP synthesis, induced by angiotensin II, counteracts water and sodium retention caused by the activated RAAS, a well-known promoter of CKD progression and poor renal prognosis [30]. When salt and water retention was irrevocably established, high levels of BNP characterized patients at pre-dialytic stages, depicting the failure of compensatory mechanisms, not sufficient to preserve a water/sodium/volume homeostasis.

High levels of BNP—precociously highlighting a volume overload—reflect a high venous return and diastolic dysfunction which (associated with renal failure) increase pulmonary venous and arterial pressures, representing the hemodynamic prelude of PH [31]. Consequently, the progressive increase of BNP and the decrease in renal function are necessarily associated with pulmonary involvement, amplifying the progression of both cardiac and kidney failure, leading to ESRD and chronic heart failure [32].

Moreover, we underlined a strict relationship between BNP, PH, and diastolic dysfunction, independently of GFR. We also identified that pulmonary artery pressure was independently associated with renal outcome. PH, increasingly recognized in patients with CKD, is induced and/or aggravated by left ventricular disorders, as well as typical CKD risk factors, such as volume overload, vascular calcification, stiffening, and severe anemia [33,34,35].

The regression of increased LVMi is possible with drugs that block the renin–angiotensin–aldosterone system, and this effect appears to be, at least in part, independent of their blood pressure-lowering effect. Whereas vitamin D did not change LVMi or impact left ventricular ejection fraction or fibrosis [36], six months of exposure to the sodium–glucose cotransporter-2 inhibitor (SGLT2i) empagliflozin resulted in reduced LVMi [37,38]. The exact mechanisms underlying SGLT2i-associated LVMi regression remain unclear, although osmotic diuresis, reductions in preload or afterload, and improved glycemic control may play a role. All these events should improve myocyte hypertrophy, myocardial steatosis, and inflammation [39]. Moreover, empagliflozin significantly improved survival in rats with monocrotaline-induced pulmonary artery hypertension while reducing mean PAPs, ventricular hypertrophy, and fibrosis [40], as well as the EMBRACE-HF trial showed that in patients with severe heart failure, empagliflozin decreased pulmonary artery pressures independent of loop diuretic therapy [41].

No patients in our cohort were treated with SLGT2i. However, although empagliflozin may be a potentially promising agent to reverse cardiac remodeling in clinical practice, further studies are needed to evaluate the physiopathological mechanism of its positive effects.

High LVMI could represent an independent prognostic factor for the decline in renal function until the start of renal replacement therapy and a risk for major cardiovascular events. Stratifying patients with stage III CKD according to LVMI levels could help clinicians to monitor patients with nephropathy more closely, providing maximal renal and heart protection. Moreover, ventricular geometry, myocardial mass, myocardial fibrosis, and renal outcomes could be the target for new drugs, such as SGLT2 inhibitors or anti-fibrotic agents.

Our study has limitations that must be mentioned. It was a single-center study, with a relative low number of patients, and confirmation in wider cohorts is indispensable to attribute general validity to our reports.

The duration of follow-up time was chosen to be relatively brief on purpose, due to the comorbidities of our cohort, their age, and the mean GFR evaluated at the enrolment phase. Notwithstanding, the primary endpoint was reached by 26% of the participants, and the statistical model was powerful enough to establish independent relationships between biomarkers and CKD progression. Moreover, because the time of follow up varied considerably among patients who reached neither of the renal endpoints, our study design is not appropriate to define a conclusive cut-off for stratifying the risk of reaching a doubling of creatinine/ESRD within defined time intervals.

However, the small number of patients included, especially in each subgroup of patients with CKD, allowed for recruiting with strict inclusion and exclusion criteria, minimizing the effect of confounding factors on cardiac structure and function. In fact, we enrolled patients without cardiac dysfunction or volume overload, without altered BNP values at the enrolment, hypothesizing that the obtained data were a consequence of renal dysfunction. Moreover, the renal function at the enrolment was established through a measured and not an estimated GFR.

## 5. Conclusions

In subjects affected by non-terminal CKD, high LVMi represents an independent predictor of the progression of renal disease. Further in-depth examinations should be undertaken to verify whether these findings can be confirmed in a longer observational period and to determine if therapeutic measures targeting the cardiac structure, such as SGLT2i and anti-fibrotic agents, i.e., finrenone, could delay the progression of renal disease. Echocardiography can help identify patients at high risk for renal disease progression, but its potential benefit in CKD populations, independently of clinical cardiac involvement, should be investigated in prospective trials.

## Figures and Tables

**Table 1:** Demographic, clinical characteristics, and echocardiographic data of studied population at baseline and during the follow-up period.

	Baseline *n* = 160	Progressor *n* = 41 (26%)	Non Progressor *n* = 119 (74%)	*p*
Age, years	71.5 ± 6.3	73.6 ± 6.8	69.5 ± 9.3	0.12
BMI	28.8 ± 5.6	28.2 ± 2.7	26.4 ± 4.1	0.42
Diabetes mellitus, *n*	68	31	37	0.34
Current smokers, *n*	38	13	25	0.13
Systolic BP, mmHg	129.8 ± 21.9	128.7 ± 26.1	130.2 ± 20.5	0.79
Diastolic BP, mmHg	73.5 ± 13.8	73.2 ± 13.7	74.3 ± 14.5	0.77
Prior CV events, *n* (%)	42 (26%)	13 (31)	29 (24)	0.36
Albumin, g/dL	3.7 ± 0.9	3.8 ± 0.3	3.9 ± 0.4	0.16
Hemoglobin, g/dL	11 ± 3.3	11.3 ± 1.2	11.5 ± 2	0.71
C-reactive protein, mg/L	0.2 (0.1–0.5)	0.5 (0.2–0.8)	0.2 (0.1–0.3)	0.82
Homocysteine, μmol/L	24.9 ± 6.4	23.2 ± 5.4	20.2 ± 5.3	0.10
Uric acid, mg/dL	6.6 ± 2.2	7.3 ± 1.9	6.6 ± 1.4	0.13
PTH, pg/mL	58.2 (28.3–98.5)	58.5 (29.2–92.1)	54.5 (27.9–99.6)	0.61
VIT -D3, pg/mL	21.3 ± 10.3	20.9 ± 9.8	22.3 ± 11.7	0.62
Total cholesterol, mg/dL	165.6 ± 40.3	176.4 ± 43.2	162.3 ± 38.8	0.09
HDL cholesterol, mg/dL	50.3 ± 12.7	50.1 ± 12.9	50.5 ± 12.9	0.95
LDL cholesterol, mg/dL	106 ± 65.2	129 ± 69	102 ± 64	0.08
Triglycerides, mg/dL	132.2 ± 70.9	136.3 ± 77.8	130.2 ± 67.8	0.66
Serum osmolality, mOsm/kg	296.6 ± 12.4	295.7 ± 12.3	298 ± 12.7	0.42
Urine osmolality, mOsm/kg	365.8 ±76.6	370.6 ±77.3	356 ±76.4	0.49
Natriuria, mEq/24 h	69 ± 28.4	70.8 ± 22.5	65.1 ± 19.9	0.57
Serum creatinine, mg/dL	2.01 ± 1.05	4.7 ± 2.4	1.7 ± 1.1	0.03
mGFR, mL/min	34.8 ± 15	19.8 ± 9.3	41.5 ± 11.8	<0.001
Albuminuria, mg/24 h	50 (10–349)	714 (91.7–1902)	32 (9.2–88.5)	0.03
Proteinuria, mg/24 h	140.5 (68.7–358.5)	384 (180–2626)	105 (65–188)	0.03
BNP, pg/mg	18.7 (10–32.2)	46.2 (30.2–65.5)	14.4 (10–20.6)	<0.001
Echocardiography parameters				
LVIDd, mm	43.8 ± 3.1	44.1 ± 3.3	43.2 ± 2.7	0.46
LVIDs, mm	39.5 ± 2.6	41.3 ± 1.9	40.6 ± 2.1	0.67
IVST, mm	12.6 ± 1.5	13.7 ± 1.8	11 ± 1.1	<0.001
LVPW, mm	9.5 ± 1.5	10.5 ± 1.1	9.1 ± 1.5	0.03
LVEF, %	63.2 ± 4.7	59.5 ± 2.7	64.6 ± 3.8	0.002
LVMi, g/m^2^	101.9 ± 12.2	115.6 ± 12.2	84.2 ± 10.1	0.008
Mitral E/A ratio, cm/s	0.87 (0.67–1.16)	0.98 (0.87–1.23)	0.84 (0.77–1.02)	0.001
PAPs, mmHg	35.8 ± 9.2	48.3 ± 2.8	31.6 ± 6.1	0.001

Abbreviations: BMI: body mass index; BP: blood pressure; HDL: high-density lipoprotein; LDL: low-density lipoprotein; mGFR: measured glomerular filtration rate; BNP: brain natriuretic peptide; PTH: par-athyroid hormone; VIT D3: 1,25 dihydroxyvitamin D3, pg/mL; LVIDd: left ventricular internal diameter end diastole; LVIDs: left ventricular internal diameter end systole; IVST: inter-ventricular septal thickness; LVPW: left ventricular posterior wall; LVEF: left ventricular ejection fraction; LVMi: left ventricular mass index; E/A: ratio of early and late diastolic velocities; PAPs: systolic pulmonary artery pressure.

**Table 2:** Univariate and multiple regression analysis of LVMi at baseline.

Variable	Partial R	β	*p*
sBP	0.45 (*p*: 0.01)	0.61	<0.001
PAPs	0.75 (*p*: 0.004)	0.56	0.01
BNP	0.32 (*p*: 0.02)	0.10	0.12
Natriuria mEq/24 h	0.79 (*p* < 0.001)	0.76	<0.001
mGFR, mL/min	−0.38 (*p*: 0.01)	−0.23	0.03
Proteinuria	0.41 (*p* < 0.001)	0.70	0.001
Hemoglobin	−0.20 (*p*: 0.02)	−0.11	0.10

Multiple R: 0.86, R2: 65%; *p*: 0.0006. β is the standardized coefficient of correlation. Abbreviations: sBP: systolic blood pressure; LVMi: left ventricular mass index; LVEF: left ventricular ejection fraction; PAPs: systolic pulmonary artery pressure; BNP: brain natriuretic peptide; mGFR: measured glomerular filtration rate.

**Table 3:** Regression analysis of the association between baseline LVMi and decline in renal function in chronic kidney disease patients.

CKD Worsening *	Crude		Model 1		Model 2		Model 3	
	β	*p*	β	*p*	β	*p*	β	*p*
LVMi	0.010	<0.001	0.010	<0.001	0.033	0.003	0.025	0.01
Age			0.060	0.26	0.13	0.26	0.10	0.31
Gender (male)			0.076	0.91	1.72	0.29	0.94	0.69
BMI					0.34	0.29	0.16	0.15
BP (yes)					0.020	0.03	0.013	0.08
Proteinuria 24 h					0.022	0.01	0.016	0.03
Diabetes (yes)					0.012	0.22	0.18	0.13
Smoking (yes)					0.060	0.10	0.024	0.34
BNP							0.006	0.04
LVEF							−0.048	0.002
CV events (yes)							0.015	0.11

* Dependent dichotomic variable revealed by doubling of serum creatinine and/or end-stage renal disease requiring dialytic therapy. β: coefficient. Abbreviations: LVMi: left ventricular mass index; BMI: body mass index; BP: blood hypertension; BNP: brain natriuretic peptide; LVEF: left ventricular ejection fraction; CV: cardiovascular.

**Table 4:** Univariate and multivariate Cox proportional hazards regression model for incidence of chronic kidney disease progression during a 1-year follow-up period.

	Univariate Analysis			Multivariate Analysis		
	HR	95% CI	*p* Value	HR	95% CI	*p* Value
Age, years	1.03	0.91–1.06	0.11			
Uric acid, mg/dL	1.02	1.01–1.04	0.03	1.03	1.01–1.10	0.02
IVST, mm	1.02	0.94–1.03	0.21			
LVPW, mm	1.01	0.92–1.04	0.11			
LVEF, %	0.95	0.87–0.98	0.01	0.96	0.92–1.01	0.08
LVMi, g/m^2^	1.06	1.03–1.11	<0.001	1.12	1.04–1.16	0.001
Mitral E/A ratio, cm/s	1.01	0.98–1.11	0.61			
PAPs, mmHg	1.07	1.02–1.13	0.003	1.09	1.03–1.16	<0.001
Creatinine, mg/dl	1.06	1.01–1.13	0.01			
mGFR, mL/min	0.83	0.74–0.96	<0.001	0.82	0.77–0.97	<0.001
Albuminuria, mg/24 h	1.03	0.96–1.02	0.14			
Proteinuria, mg/24 h	1.05	1.01–1.07	0.03	1.08	1.03–1.14	<0.001
BNP, pg/mg	1.06	1.01–1.10	0.01	1.07	1.03–1.10	0.02

Abbreviations: IVST: inter-ventricular septal thickness; LVPW: left ventricular posterior wall; LVEF: left ventricular ejection fraction; LVMi: left ventricular mass index; E/A: ratio of early and late diastolic velocities; PAPs: systolic pulmonary artery pressure; mGFR: measured glomerular filtration rate; BNP: brain natriuretic peptide.

## Data Availability

The data presented in this study are available on request from the corresponding author.
